# Moxibustion for treating patients with hyperlipidemia: A systematic review and meta-analysis protocol

**DOI:** 10.1097/MD.0000000000018209

**Published:** 2019-11-27

**Authors:** Qin Yao, Xinyue Zhang, Yueping Huang, Hao Wang, Xin Hui, Baixiao Zhao

**Affiliations:** Beijing University of Chinese Medicine, Beijing, China.

**Keywords:** complementary medicine, hyperlipidemia, moxibustion, protocol

## Abstract

**Background:**

Hyperlipidemia has been a root cause of atherosclerosis, which leads to a high risk to serious cardio-cerebrovascular disease. Many trials have reported that moxibustion therapy is effective in lowering blood lipid levels when treating hyperlipidemia. The aim of this systematic review is to assess the effectiveness and safety of moxibustion therapy for hyperlipidemia.

**Methods:**

Two reviewers will electronically search the following databases: the Cochrane Central Register of Controlled Trials (CENTRAL); PubMed; EMBASE; China National Knowledge Infrastructure (CNKI); Chinese Biomedical Literature Database (CBM); Chinese Scientific Journal Database (VIP database); and Wan-Fang Database from the inception, without restriction of publication status and languages. Additional searching including researches in progress, the reference lists and the citation lists of identified publications. Study selection, data extraction, and assessment of study quality will be performed independently by 2 reviewers. Changes of blood lipid levels from baseline to the end of the treatment, including low-density lipoprotein cholesterol (LDL-C) level, total cholesterol (TC) level, triglycerides (TG) level and high-density lipoprotein cholesterol (HDL-C) level will be assessed as the primary outcomes. Quality of life, long-term effect and safety will be evaluated as secondary outcomes. If it is appropriate for a meta-analysis, RevMan 5.3 statistical software will be used; otherwise, a descriptive analysis will be conducted. Data will be synthesized by either the fixed-effects or random-effects model according to a heterogeneity test. The results will be presented as risk ratio (RR) with 95% confidence intervals (CIs) for dichotomous data and weight mean difference (WMD) or standard mean difference (SMD) 95% CIs for continuous data.

**Results:**

This study will provide a comprehensive review of the available evidence for the treatment of moxibustion with hyperlipidemia.

**Conclusions:**

The conclusions of our study will provide an evidence to judge whether moxibustion is an effective and safe intervention for patients with hyperlipidemia.

**Ethics and dissemination:**

This systematic review will be disseminated in a peer-reviewed journal or presented at relevant conferences. It is not necessary for a formal ethical approval because the data are not individualized.

**Trial registration number:**

PROSPERO CRD42019130545.

## 1 Introduction

### 1.1 Description of the condition

Hyperlipidemia is a condition with excess lipids in the blood, including high total cholesterol (TC), high triglycerides (TG), and elevated low-density lipoprotein cholesterol (LDL-C).^[1,2]^ An elevated level of cholesterol is a root cause of atherosclerosis, leading to coronary heart disease, stroke, and myocardial infarction.^[3–5]^ Multiple meta-analyses and individual randomized controlled trials (RCTs) have demonstrated that atherogenic cholesterol, especially LDL-C, is the most important risk factor for atherosclerotic cardiovascular disease (ASCVD) events.^[6–11]^ The prevalence of the adults in the United States of high TC, high LDL-C, high TG, and low high-density lipoprotein cholesterol(HDL-C) are 11.0%, 28.5%, 24.2%, and 17%, respectively.^[12]^ The prevalence of hyperlipidemia may differ with ages, genders, races, resident regions, and socioeconomic status, etc.^[12–14]^ The management of hyperlipidemia is aimed at reducing elevated levels of atherogenic cholesterol, which has been verified to decrease the incidence of ASCVD.^[15]^ A heart-healthy lifestyle across the life course has been emphasized as the basis of therapy and statins are the cornerstone of lipid-lowering drugs.^[16,17]^ Other drugs include ezetimibe, bile acid sequestrants, proprotein convertase subtilisin/kexin type 9 (PCSK9) inhibitors, fibrates, niacin, etc. The most frequent statin-associated side effects are muscle symptoms, of which subjective myalgias with normal creatine kinase most commonly described and rhabdomyolysis is the most severe one, other side effects are not frequently reported, such as increased risk of incident diabetes mellitus, transaminase elevation.^[16–18]^

### 1.2 Description of the intervention

Moxibustion is an external therapy using burning herbal preparations containing Artemisia vulgaris (mugwort) to produce a warm sensation on the acupoints or areas of body surface.^[19,20]^ Moxibustion, originated as early as the clan commune period of the primitive society in China, is an important part of traditional Chinese medicine (TCM) and widely used in the treatment and prevention of many disorders.^[21–23]^ Moxibustion techniques can be divided into moxa-stick moxibustion, moxa-cone moxibustion or moxibustion with moxibustioner (a device for moxibustion), whilst moxa-cone moxibustion includes direct moxibustion and indirect moxibustion. Indirect moxibustion is achieved by placing insulating materials such as salt, monkshood cake, sliced ginger or garlic between the skin and a burning moxa cone. Moxibustion is associated with some potential adverse events, such as allergy, burn, coughing, according to 2 systematic reviews.^[24,25]^

### 1.3 How the intervention might work

In TCM, moxibustion is believed to take effect by warming the body's meridians and promoting better circulation of vital energy (in Chinese called qi and blood). In Western medicine, the mechanism of moxibustion is yet not clear. And five main factors were possibly involved in the process, including the function of moxa and its components, warming effects (moxa heat sensation), radiation effects, moxa smoke, and aromatherapy.^[26]^ Studies in animals and humans signified that moxibustion is effective in reducing TC, TG, and LDL-C level for treating hyperlipidemia.^[27–29]^

### 1.4 Why it is important to do this review

Although statins have been found to be highly effective in the management of hyperlipidemia, resistance, and intolerability to side effects will continue to be a stumbling block for certain patients. Researchers have developed a large number of clinical studies in moxibustion regulating lipid profile of hyperlipidemia patients. So far, there has been no systematic review published in English assessing moxibustion for hyperlipidemia. It is therefore important to assess whether moxibustion is a good choice for hyperlipidemia patients, and whether it is as effective to be a supplement therapy to conventional therapies. In this article, we present the protocol of our proposed systematic review in moxibustion for hyperlipidemia patients.

### 1.5 Objectives

To systematically evaluate the effectiveness and safety of moxibustion therapy for hyperlipidemia patients.

## 2 Methods and analysis

This protocol has been drafted under the guidance of the Preferred Reporting Items for Systematic Reviews and Meta-analysis Protocols (PRISMA-P) and Cochrane handbook for systematic reviews of interventions.^[30,31]^ This protocol has been registered on the PROSPERSO website (https://www.crd.york.ac.uk/PROSPERO/) and any important protocol amendments will be recorded.

### 2.1 Inclusion criteria for study selection

#### 2.1.1 Types of studies

Randomized controlled trials (RCTs) will be included, without restrictions on publication status. Quasi-randomized trials will not be included.

#### 2.1.2 Types of participants

Adult patients with hyperlipidemia, regardless of sex, race, or educational and economic status.

#### 2.1.3 Types of interventions

All kinds of moxibustion therapy will be included, such as moxa-stick moxibustion, moxa-cone moxibustion or moxibustion with moxibustioner (a device for moxibustion). We excluded moxa needle therapy, which consists of inserting a needle into an acupoint and wrapping the end of the needle in an ignited moxa, because this treatment method involves acupuncture and makes it impossible to evaluate whether the treatment effect is due only to moxibustion.

#### 2.1.4 Types of comparisons

The following treatment comparisons will be investigated.

1. Moxibustion compared with no treatment.
2. Moxibustion compared with no treatment or sham moxibustion.
3. Moxibustion compared with conventional western medicine.
4. Moxibustion in addition to conventional western medicine compared with the same medicine.

Most sham moxibustion uses sham device resembles the real moxibustioner except putting some components in to insulate the heat and smoke produced by the burning moxa and prevent them from radiating to the skin.^[32]^ We will exclude RCTs in which one form of moxibustion was only compared with another form of moxibustion or a different type of complementary therapy (eg, Chinese herbal medicine or acupuncture).

#### 2.1.5 Types of outcome measures

*Primary outcomes:* The primary outcomes are changes of blood lipid levels from baseline to the end of the treatment, including LDL-C level, TC level, TG level and HDL-C level. Blood lipid levels, especially LDL-C level are directly related to therapy effect of hyperlipidemia. The included studies should contain at least one of the primary outcomes. And the time point of the effect measures was at the end of treatments.

*Secondary outcomes:* The following secondary outcomes will be assessed:

1. Quality of life will be measured at the end of the treatments.
2. Long-term effect will be measured from baseline to the last available follow-up.
3. Adverse events will be measured throughout the studies.

### 2.2 Search methods for identification of studies

#### 2.2.1 Electronic searches

The following databases from the inception to October 2019 will be searched by 2 independent reviewers, without restriction to publication status and languages: the Cochrane Central Register of Controlled Trials (CENTRAL); PubMed; EMBASE; China National Knowledge Infrastructure (CNKI); Chinese Biomedical Literature Database (CBM); Chinese Scientific Journal Database (VIP database); and Wan-Fang Database. A search strategy for PubMed database, which is established according to the Cochrane handbook guidelines, is shown in Table 1. Similar search strategies will be applied for the other databases. Before this review completed, the 2 reviewers will conduct the searching once again to ensure the latest studies could be included.

**Table 1:**
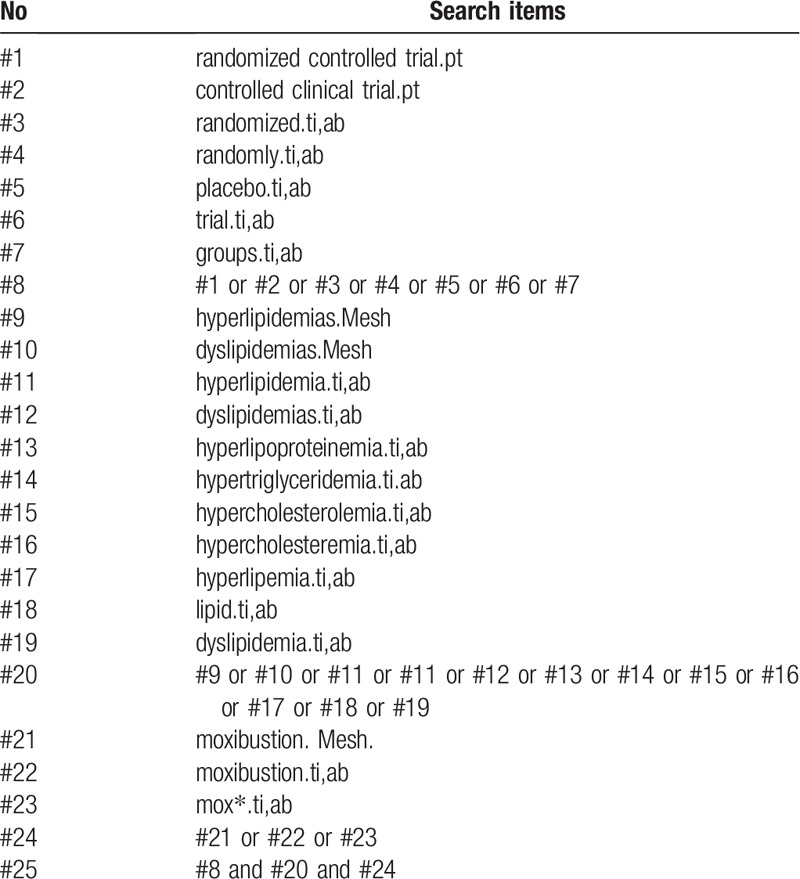
Search strategy used in PubMed.

#### 2.2.2 Searching other resources

Besides, electronic sources for relevant researches in progress will also be searched, including Clinicaltrials.gov (http://www.clinicaltrials.gov) and the World Health Organization International clinical trials registry search portal (http://apps.who.int/trialsearch/). Additionally, the citation list will be retrieved in Web of Science. Besides, the reference lists of those studies meeting the inclusion criteria and relevant systematic reviews will also be identified for additional relevant studies.

### 2.3 Data collection and analysis

#### 2.3.1 Selection of studies

**Figure 1:**
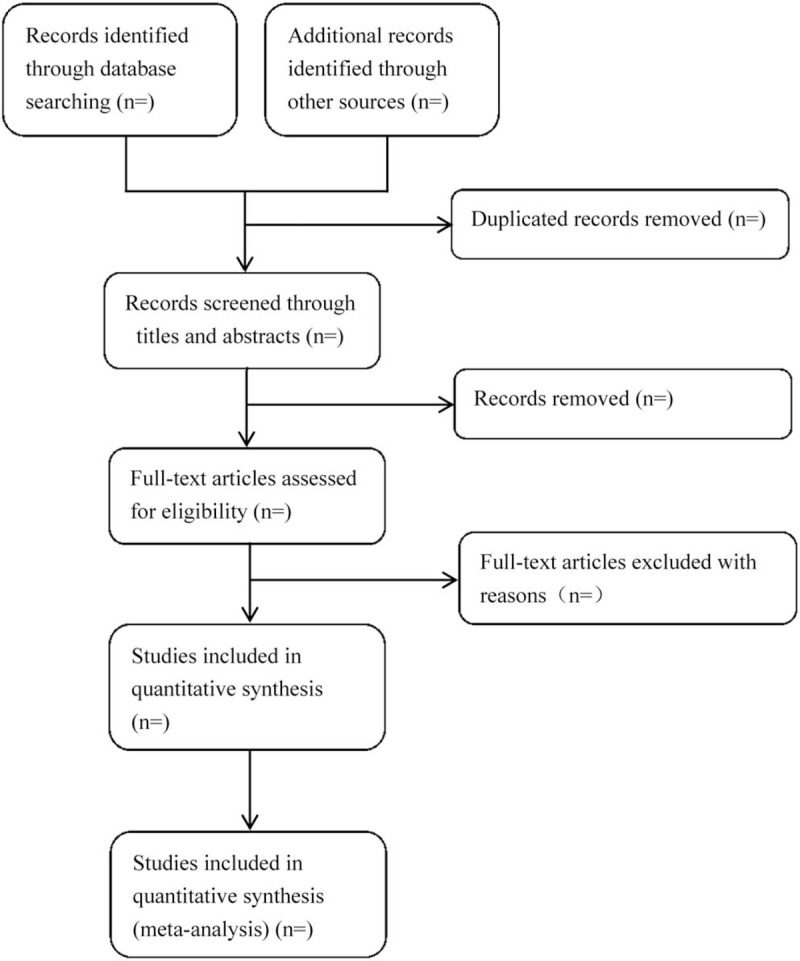
Flow diagram of the study selection process.

We plan to conduct this systematic review between 30 September 2018 and 30 June 2020. All reviewers have undergone a training to ensure a basic understanding of the background and purpose of the review. After electronic searching, the records will be uploaded to a database set up by EndNote software (V.X7). Records selected from other sources will also be moved to the same database. Two reviewers (YH and XZ) will independently screen the titles, abstracts, and keywords of all retrieved studies and decide which trials meet the inclusion criteria. We will obtain the full text of all possibly relevant studies for further assessment. Excluded studies will be recorded with explanations. Any disagreements will be resolved by discussion between the 2 reviewers (YH and XZ) and the third author (BZ) for arbitration when necessary. We will contact reviewers of trials for clarification when necessary. The study flow diagram is shown in Figure 1.

### 2.4 Data extraction and management

A unified data extraction form will be designed by all of the reviewers and its applicability will be tested in a small scope of trials by two reviewers (XH and HW). They will then independently extract data in the following domains: general information, participants, methods, interventions, outcomes, results, adverse events, conflicts of interest, ethical approval, and other information. Any disagreement will be discussed between the 2 reviewers, and further disagreements will be arbitrated by the third author (BZ).

### 2.5 Assessment of risk of bias in included studies

The risk of bias will be assessed by two reviewers (XH and HW) with the Cochrane Collaboration's tool for risk of bias assessment. The risk of bias in included studies will be evaluated according to the following aspects: sequence generation, allocation sequence concealment, blinding of participants and personnel and outcome assessors, incomplete outcome data, selective outcome reporting, and other sources of bias. The assessments will be classified into three levels: low risk, high risk, and unclear risk.

### 2.6 Measures of treatment effect

RevMan V.5.3 will be used for data analysis and quantitative data synthesis. For continuous data, we will use standard mean difference (SMD) to measure the treatment effect with 95% confidence intervals (CIs). For dichotomous data, a risk ratio (RR) with 95% CIs for analysis will be adopted.

### 2.7 Unit of analysis issues

Data from studies with parallel-group will be included for meta-analysis. For randomized cross-over trials, only the first phase data will be included. In these trials, participants are individually randomized to two intervention groups, and a single measurement of each outcome from each participant is collected and analyzed.

### 2.8 Dealing with missing data

We will try to contact the first or corresponding authors of the included studies by telephone or email to retrieve missing or insufficient trial data. If missing data are unavailable, we will make an assumption using the terms “missing at random” and “not missing at random” to represent different scenarios, which is recommended in the Cochrane Handbook.^[33]^ For the data “missing at random”, only the available data will be analyzed. For the data “not missing at random”, we will displace the missing data with replacement values and a sensitivity analysis will be used to determine whether the results are inconsistent.

### 2.9 Assessment of heterogeneity

We will use the *I*^2^ statistic for quantifying inconsistencies among the included studies. When the *I*^2^ value is less than 50%, the study will not be considered to have heterogeneity. When the *I*^2^ value exceeds 50%, significant statistic heterogeneity exists among the trial and meta-analysis will not be performed. Subgroup analysis will be conducted to ascertain possible causes.

### 2.10 Assessment of reporting biases

We will use funnel plots to detect reporting biases and small-study effects. If more than 10 studies are included in the meta-analysis, we will conduct a test for funnel plot asymmetry using Egger method. All eligible trials will be included, regardless of their methodological quality.

### 2.11 Data synthesis

Data synthesis will be performed with RevMan V.5.3 software from the Cochrane Collaboration when it is possible to conduct a meta-analysis. Both clinical homogeneity and methodological homogeneity will be assessed by professional assessors. The dichotomous data will be combined by using risk ratio (RR) and continuous data will be integrated by using SMD with 95% CI. If there is no significant heterogeneity detected, the fixed effect model will be applied for data synthesis; otherwise, the random-effects model will be conducted. If the situation that quantitative synthesis is not appropriate, such as insufficient trials or unidentified significant heterogeneity, a systematic narrative synthesis will be applied to describe the characteristics and findings of the included trials.

### 2.12 Sensitivity analysis

Sensitivity analysis will be conducted to validate the robustness of the primary results. We will exclude certain trials by a re-evaluation of methodological quality, study types, sample size, missing data or other possible factors. Careful interpretations will be employed for sensitivity analysis if differ substantially.

### 2.13 Grading the quality of evidence

The Grading of Recommendations Assessment, Development and Evaluation (GRADE) working group methodology will be applied for the quality of evidence for all outcomes.^[34]^ Six domains will be assessed, containing risk of bias, consistency, directness, precision, publication bias and additional points. The assessments will be categorized into 4 levels: high, moderate, low, or very low.

### 2.14 Subgroup analysis

If data are available, a subgroup analysis will be performed based on the type of moxibustion intervention (moxibustion with moxa stick, direct moxibustion, indirect moxibustion, etc) because this is the main factor causing heterogeneity. Additionally, the type of hyperlipidemia will also be considered.

## 3 Discussion

Hyperlipidemia are associated with many diseases including coronary heart disease, stroke, and myocardial infarction etc, which are leading cause of mortality and morbidity worldwide, according to the World Health Organization.^[35]^ Various guidelines emphasize the importance of managing the lipid profile.^[15–17]^ Though statins have been recommended to be the first-line drugs for the management of hyperlipidemia, the side effects and economic burden are still concerned.^[36,37]^ Therefore, it is meaningful to resort to complementary approach to manage hyperlipidemia.

Researchers have developed a large number of clinical studies in moxibustion regulating lipid profile of hyperlipidemia patients. Nevertheless, no systematic review related to moxibustion for hyperlipidemia has been published in English so far. In this paper, we present a protocol for a systematic review of moxibustion for hyperlipidemia patients. Thus, this systematic review will analyze current clinical evidence about effectiveness and safety of moxibustion for hyperlipidemia patients. We hope this review will facilitate clinicians when making decisions.

## Author contributions

**Conceptualization:** Qin Yao

**Methodology and project conduct:** Qin Yao, Xinyue Zhang, Yueping Huang, Hao Wang, Xin Hui, Baixiao Zhao

**Supervision:** Baixiao Zhao

**Writing – original draft:** Qin Yao

**Writing – review & editing:** Qin Yao, Xinyue Zhang, Yueping Huang, Hao Wang, Xin Hui, Baixiao Zhao
